# Topological transition at midlife in the physiological network of Mexican women and divergent amplitude of topological change between sexes: allostatic load biomarkers across adult life

**DOI:** 10.3389/fphys.2026.1982421

**Published:** 2026-10-21

**Authors:** Julian B. Uriarte-Ortiz, Antonio Barajas-Martínez, Ruth M. López-Mayorga, Raúl Sampieri-Cabrera

**Affiliations:** 1 Department of Physiology, Faculty of Medicine, National Autonomous University of Mexico, Mexico, Mexico; 2 Instituto Politécnico Nacional, Escuela Superior de Medicina, Sección de Estudios de Posgrado e Investigación, Mexico, Mexico; 3 Instituto Tecnológico y de Estudios Superiores de Monterrey, México, Mexico; 4 Centro de Ciencias de la Complejidad, Universidad Nacional Autónoma de México, México, Mexico

**Keywords:** allostatic load biomarkers, dyslipidemia, midlife transition, physiological network topology, sexual dimorphism

## Abstract

**Introduction:**

Additive allostatic load indices treat cardiometabolic biomarkers as independent Q6 units and discard their covariation. Correlation networks recover it, yet previous physiological network studies were restricted to strictly healthy participants or specific diseases, leaving undescribed how topology changes across adult life in the general population.

**Methods:**

We analyzed 7,999 adults (5,058 women, 2,941 men) pooled from the Mexican National Health and Nutrition Surveys (ENSANUT) of 2016, 2020 and 2021, stratified by sex and five age groups (20–91 years) and, in an exploratory analysis, by dyslipidemia. For each stratum we built graphs of 22 anthropometric, hemodynamic and biochemical variables from Spearman correlations retained at p < 0.05 and averaged density, transitivity, mean distance and Louvain modularity over 100 subsamples of 79% of the participants.

**Results:**

Female topology followed two phases. Between the 20–29 and 40–49 age groups, density fell from 0.47 to 0.25 and transitivity from 0.70 to 0.50, whereas mean distance rose from 1.61 to 2.32 and modularity from 0.40 to 0.56 as blood pressure variables separated into a distinct module; all four then plateaued through the oldest group. Univariate biomarker trajectories changed gradually over the same ages. Men showed no such transition, with density between 0.34 and 0.42 across all strata, a range narrower than each female step during the first phase, and modularity rising only from 0.37 to 0.45 between the two youngest groups. The female decline was already present between the 20–29 and 30–39 groups, at ages preceding the menopausal transition. Edges persisted in graphs rebuilt from the 12 directly measured variables, and stratification by dyslipidemia, defined by three nodes of the network, did not fully account for the age trends.

**Discussion:**

These cross-sectional graphs describe covariation across individuals, and their reading as within-person regulation requires longitudinal confirmation. A male transition before age 20 and survivorship selection remain competing, testable explanations for male topological stability. The results extend to adult life the sexual dimorphism described in physiological networks of healthy young adults and locate in women a reorganization, completed by midlife, that univariate analyses did not reveal.

## 1 Introduction

Human physiology is a complex system whose variables couple to one another to sustain homeostasis, and the topology of these couplings shifts with health status, age and sex (Bashan et al., 2012; Ivanov, 2021). Homeostasis arises from the balance between robustness, which preserves the functional identity of the system, and adaptability, which allows it to respond to environmental changes; thus, loss of this balance leads to disease (Fossion et al., 2018). The allostatic load model describes the cumulative counterpart of the same process, since repeated perturbations, injuries and wear of the system end up being reflected in blood pressure, glucose metabolism, lipid profile, protein synthesis and renal function (McEwen and Stellar, 1993; McEwen, 2007; Chrousos, 2009). These functional compartments are usually examined through point-in-time measurements, and the state of the system is summarized as an operationalized threshold of single or multiple biomarkers crossing population based risk quantiles (Seeman et al., 1997); additive indices have shown mortality prediction and functional decline with notable consistency (Juster et al., 2010) at the cost of treating biomarkers as independent units and discarding the information contained in their coordination. Following that literature, we refer to these variables as allostatic load biomarkers, which correspond to secondary outcomes of primary neuroendocrine and immune mediators (McEwen, 2007; Juster et al., 2010). We describe their position within the physiological network without estimating causal effects, and no mediation analysis was performed.

Inference networks built from correlation matrices in narrow-age cohorts recover this relational information and describe systemic properties that isolated variables alone cannot show (Barajas-Martínez et al., 2020). This approach yields a model of the system that has revealed age transitions in the roles played by physiological variables within the network (Barajas-Martínez et al., 2020), and in healthy young adults, sex-dependent topology differences such as the robustness of the physiological network in females. These descriptive models become predictive when simulated network attacks are used to anticipate the response of the network. For instance, the male network shows greater modularity and small-world index at the cost of greater vulnerability (Barajas-Martínez et al., 2021). Network robustness predicts easier systemic propagation of a perturbation whereas network adaptability predicts easier disruption of the system interactions. These predictions held for women with achalasia (Furuzawa-Carballeda et al., 2024) and men with acute COVID-19 (Barajas-Martínez et al., 2022).

The loss of complexity with aging and the increase in multisystem dysregulation, measurable as statistical distance from the young profile (Cohen et al., 2013), suggest that this architecture should degrade across the lifespan. The temporal form of this degradation remains undescribed, since previous network studies were restricted either to young subjects under strict health criteria, or particular health states of interest. It is unknown how topology evolves in the general population, where a high metabolic burden coexists with aging.

To make matters more complex, our own physiology varies throughout time. The secretion of hormones related to the hypothalamic-pituitary-adrenal axis is varied across the lifespan and follows different patterns between women and men, deeply affecting overall metabolism (Kudielka and Kirschbaum, 2005; Bale and Epperson, 2015). Menopause is a physiological transition that affects lipid metabolism and vascular regulation (Matthews et al., 2009; El Khoudary et al., 2020). Not to mention the effect of diseases, whereas for example, dyslipidemia, through alterations in metabolic pathways promotes visceral lipolysis and hepatic overproduction of lipoproteins (Rosmond and Björntorp, 2000; Kyrou and Tsigos, 2009).

This study aimed to describe the topology of allostatic load on the physiological network across adult life in the Mexican population, stratified by sex, age group and the presence of dyslipidemia.

## 2 Materials and methods

### 2.1 Ethics statement

This study is a secondary analysis of publicly available data by the Mexican government conducted under project PAPIIT DGAPA-UNAM code IA301325 in accordance with current regulation and ethics committee approval. All data were collected by trained professionals and all participants signed a written informed consent form and attained ethical compliance according to Mexican national standards (Romero-Martínez et al., 2017; Romero-Martínez et al., 2021a; Romero-Martínez et al., 2021b).

### 2.2 Databases

The present work comprises the analyses of anthropometric and biochemical data from a nation-wide sample in Mexico from 3 different years: 2016, 2020 and 2021. The three surveys belong to the same national program and share a probabilistic household design and similar procedures for questionnaires, anthropometry, blood pressure and blood sampling, applied by trained personnel (Romero-Martínez et al., 2017; Romero-Martínez et al., 2021a; Romero-Martínez et al., 2021b). They differ in fieldwork period, sample size and regional allocation of the sample, and the 2020 survey was conducted during the COVID-19 pandemic (Romero-Martínez et al., 2021a). In total, all bases accounted for 45,532 individuals. The unit of analysis is the individual participant. Survey weights were not applied, so the estimates describe the analytic sample and are not population estimates. Participants with incomplete data (≥20% of missing variables) were removed from analysis and only data from 7,999 participants were included (**Figure 1**). Because the three surveys differed in the variables they measured and in how some of them were measured, only variables included in all three datasets entered the final analysis, resulting in 12 directly measured variables, from which ten variables were derived: body mass index (BMI), mean blood pressure, pulse blood pressure, low-density lipoprotein cholesterol (LDL-C), very low-density lipoprotein cholesterol (VLDL-C), martin factor, estimated glomerular filtration rate (eGFR), METS-IR, iHOMA2-B and iHOMA2-S. The first seven were calculated using standard formulas, while iHOMA2-B and iHOMA2-S were calculated using Hill’s model (Hill et al., 2013) and METS-IR was calculated according to Bello-Chavolla’s formula: Ln((2*Glucose) +Triglycerides) * Body Mass Index (BMI))/(Ln (High-density Lipoprotein Cholesterol (HDL-C))) (Bello-Chavolla et al., 2018).

**Figure 1:**
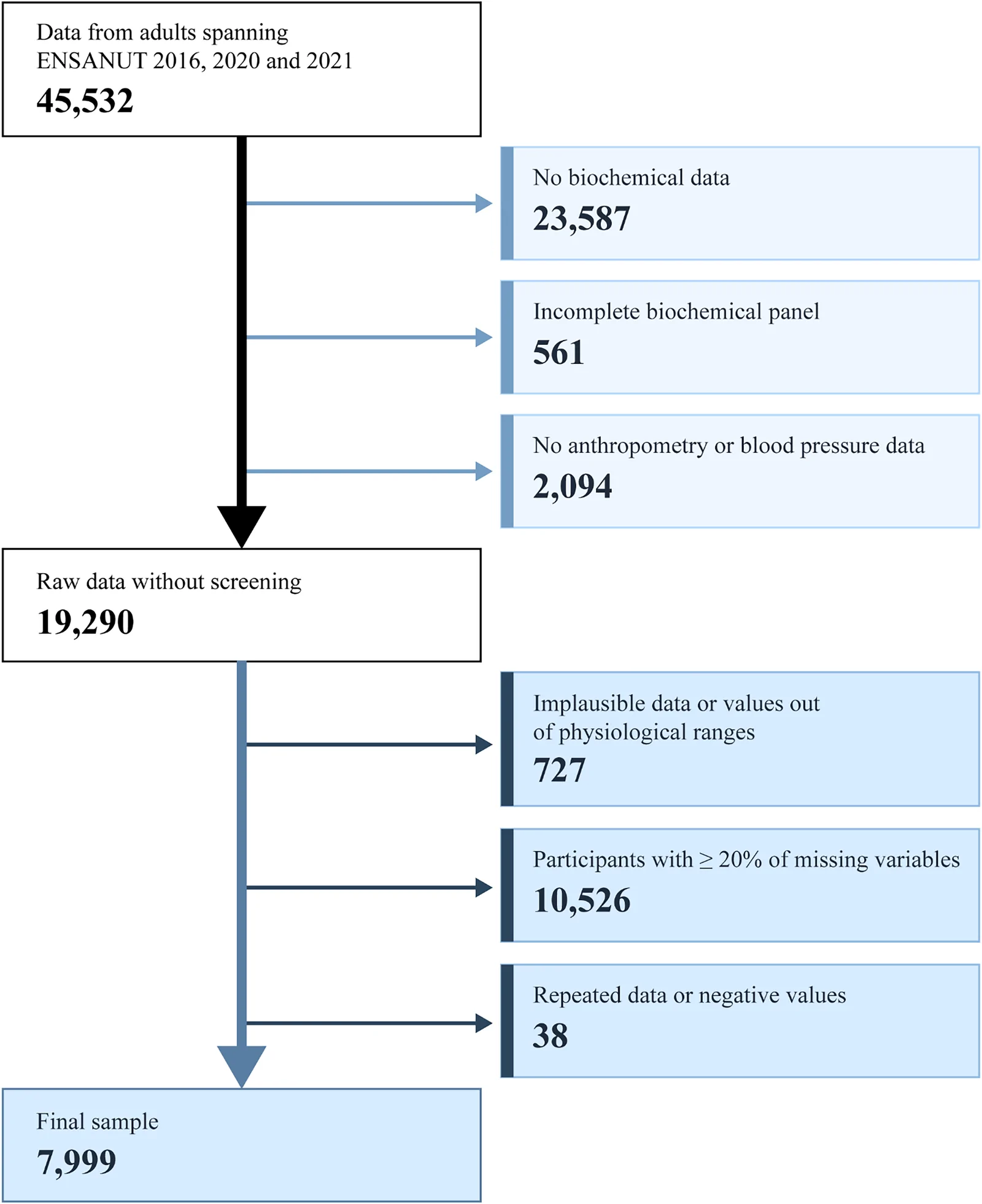
STROBE flow chart for retrospective observational studies.

### 2.3 Data processing

Databases were constructed manually in Excel (Microsoft Corporation) and validated at random as quality control. All variables except height departed from normality (**Tables 1**, **2**), such that the median value was considered the best measure of the distribution center, and the interquartile range was considered the best representation of dispersion (**Tables 1**, **2**). Implausible data (impossible values or mistakes in the native datasets) were removed and outliers within the expected ranges of biological variability were retained for analysis (**Figure 1**). The total sample was divided into five groups of increasing age (**Tables 1**, **2**), each spanning about a decade of life. In order to keep the group sizes as similar as possible for comparison, we modified the last two groups, with adults in their fifties including data from people aged 60 and 61 years old, and the final group including all adults from 62 to 91 years old. Descriptive data statistics such as normality tests, medians, interquartile range, chi-squared tests and non-parametric one-way Anova were conducted in Jamovi 2.7.38 (Sydney, Australia).

**Table 1:** Descriptive statistics of all women.

Women						
Variables	20-29 (n=971)	30-39 (n=1110)	40-49 (n=1118)	50-61 (n=998)	62-91 (n=861)	P value*
Weight (kg)	63.3 (22.5)^a^	68.3 (18.3) ^b^	70.3 (17.9) ^b^	69.8 (18.4) ^b^	63.0 (17.2) ^a^	<0.001
Height (cm)	156 (8.6)$ ^a^	154 (8.4) ^b^	153 (8.9)$ ^b^	152 (8.5)$ ^c^	148 (8.3) ^d^	<0.001
BMI (Kg/m^2^)	26.5 (7.5) ^a^	28.9 (7.1) ^b^	30.0 (6.9) ^c^	30.2 (7.0) ^c^	28.7 (6.7) ^b^	<0.001
Systolic Blood Pressure (mmHg)	104 (13.5) ^a^	109 (15.6) ^b^	115 (20.5) ^c^	124 (24.0) ^d^	132 (29.2) ^e^	<0.001
Diastolic Blood Pressure (mmHg)	68 (12.0) ^a^	71 (12.3) ^b^	74 (14.5) ^c^	75.3 (15.0) ^c^	72.0 (14.9) ^b^	<0.001
Mean Blood Pressure (mmHg)	79 (11.5) ^a^	83 (12.3) ^b^	87 (15.8) ^c^	91 (16.3) ^d^	92 (17.8) ^d^	<0.001
Pulse Pressure (mmHg)	36 (9.3) ^a^	37 (10.5) ^b^	40 (13.0) ^c^	47 (16.3) ^d^	60.0 (24.0) ^e^	<0.001
Glucose (mg/dL)	87 (13.2) ^a^	92 (14.0) ^b^	95 (17.8) ^c^	98 (30.9) ^d^	98 (29.0) ^d^	<0.001
Insulin (μUI/mL)	9.6 (9.2) ^a^	10.1 (9.5) ^a^	10.1 (8.9) ^a^	10.4 (9.4) ^a^	9.5 (8.8) ^a^	0.060
HbA1c (%)	5.1 (0.5) ^a^	5.3 (0.5) ^b^	5.5 (0.6) ^c^	5.7 (1.0) ^d^	5.8 (1.0) ^d^	<0.001
iHOMA2-B (%)	104 (63.2) ^a^	98.8 (57.7) ^b^	91.2 (54.6) ^b^	84.0 (58.8) ^c^	77.5 (57.2) ^c^	<0.001
iHOMA2-S (%)	94.7 (87.3) ^a^	87.9 (78.3) ^a,b^	86.8 (69.0) ^a,b^	83.7 (74.7) ^b^	89.0 (84.4) ^a,b^	<0.001
METS-IR	40.6 (15.0) ^a^	46.0 (14.2) ^b,d^	47.9 (13.2) ^b,c^	47.9 (13.8) ^c^	45.6 (13.1) ^d^	<0.001
Cholesterol (mg/dL)	164 (44) ^a^	172 (44) ^b^	187 (49) ^c^	199 (54) ^d^	191 (49) ^c^	<0.001
Triglycerides (mg/dL)	120 (87) ^a^	147 (99) ^b^	159 (107) ^c^	173 (111) ^d^	166 (104) ^c,d^	<0.001
HDL-C (mg/dL)	43.0 (13.6) ^a^	41.0 (13.0) ^c^	43.0 (13.0) ^a,b^	44.0 (13.1) ^b,d^	44.0 (15.0) ^d^	<0.001
LDL-C (mg/dL)	98.0 (35.2) ^a^	104.0 (33.0) ^b^	112.0 (38.0) ^c^	122.0 (42.4) ^d^	115.0 (40.6) ^c^	<0.001
VLDL-C (mg/dL)	22.6 (42.0) ^a^	27.6 (35.2) ^b^	32.8 (56.1) ^c^	37.0 (63.5) ^d^	30.5 (55.8) ^c^	<0.001
Martin Factor	5.5 (1.4) ^a^	5.9 (1.5) ^b^	6.0 (1.4) ^b^	5.9 (1.3) ^b^	6.0 (1.3) ^b^	<0.001
Albumin (g/dL)	4.3 (0.4) ^a^	4.2 (0.4) ^b^	4.1 (0.4) ^c^	4.2 (0.4) ^d^	4.1 (0.4) ^c^	<0.001
Creatinine (mg/dL)	0.6 (0.1) ^a^	0.6 (0.1) ^a^	0.6 (0.1) ^a^	0.6 (0.1) ^b^	0.6 (0.2) ^c^	<0.001
eGFR (ml/min/1.73 m2)	143 (14) ^a^	132 (13) ^b^	124 (13) ^c^	112 (13) ^d^	99 (15) ^e^	<0.001

Median values and interquartile range (in parentheses) are shown. $ indicates normal distribution for such variable and age group. *Kruskal-Wallis test. a, b, c, d, e indicate different levels of significance according to Dwass-Steel-Critchlow-Fligner pairwise comparisons.

**Table 2:** Descriptive statistics of all men.

Men						
Variables	20-29 (n=581)	30-39 (n=518)	40-49 (n=559)	50-61 (n=605)	62-91 (n=678)	P value*
Weight (kg)	73.9 (22.5) ^a^	77.8 (20.5) ^b^	77.4 (19.4) ^b^	76.5 (18.3) ^a,b^	70.4 (17.7) ^c^	<0.001
Height (cm)	169 (9.45)$ ^a^	166 (9.1)$ ^b^	166 (9.5) ^b,c^	165 (9.5)$ ^c^	162 (9.5) ^d^	<0.001
BMI (Kg/m^2^)	26.4 (7.2) ^a^	27.9 (6.7) ^b^	28.0 (5.8) ^b^	28.0 (5.9) ^b^	26.4 (5.7) ^a^	<0.001
Systolic Blood Pressure (mmHg)	118 (17.0) ^a^	121 (16.2) ^b^	122 (19.7) ^b^	127 (20.9) ^c^	131 (26.0) ^d^	<0.001
Diastolic Blood Pressure (mmHg)	72 (14.3) ^a^	75 (14.0) ^b^	77 (14.0) ^b^	77 (14.3) ^b^	72 (15.3) ^a^	<0.001
Mean Blood Pressure (mmHg)	87 (13.9) ^a^	91 (14.1) ^b^	92 (16.1) ^b,c^	94 (15.7) ^c^	91 (17.5) ^b,c^	<0.001
Pulse Blood Pressure (mmHg)	45 (12.3) ^a^	44 (10.7) ^a^	45 (12.3) ^a^	49 (13.7) ^b^	58 (20.3) ^c^	<0.001
Glucose (mg/dL)	88 (12.0) ^a^	91 (12.8) ^b^	93 (16.0) ^c^	98 (24.0) ^d^	96 (22.4) ^d^	<0.001
Insulin (μUI/mL)	7.9 (8.1) ^a^	7.8 (9.2) ^a^	8.3 (8.5) ^a^	8.1 (8.5) ^a^	6.5 (6.9) ^b^	<0.001
HbA1c (%)	5.2 (0.4) ^a^	5.3 (0.4) ^b^	5.4 (0.5) ^c^	5.6 (0.7) ^d^	5.7 (0.8) ^d^	<0.001
iHOMA2-B (%)	90.5 (63.2) ^a^	87.3 (60.1) ^a,b^	83.8 (61.6) ^b^	70.4 (56.6) ^c^	64.2 (51.8) ^c^	<0.001
iHOMA2-S (%)	114.0 (114.0) ^a,b^	112.0 (112.0) ^b^	105.0 (106.0) ^b^	106.0 (106.0) ^b^	129.0 (125.0) ^a,c^	<0.001
METS-IR	41.6 (14.2) ^a^	45.4 (14.1) ^b^	46.7 (12.9) ^b^	45.9 (13.3) ^b^	43.5 (11.8) ^c^	<0.001
Cholesterol (mg/dL)	171 (45) ^a^	179 (49) ^b,c^	185 (49) ^b^	185 (50) ^b^	178 (47) ^c^	<0.001
Triglycerides (mg/dL)	147 (113) ^a^	184 (132) ^b^	184 (148) ^b^	166 (131) ^b^	142 (109) ^a^	<0.001
HDL-C (mg/dL)	41.0 (13.2) ^a^	40.0 (12.0) ^a.c^	39.0 (13.4) ^b,c^	40.0 (12.0) ^a,c^	39.0 (12.0) ^b,c^	0.27
LDL-C (mg/dL)	102.0 (36.0) ^a^	109.0 (41.9) ^b,c^	110.0 (39.0) ^b,c^	113.0 (40.0) ^c^	107.0 (39.6) ^a,b^	<0.001
VLDL-C (mg/dL)	31.3 (62.3) ^a^	37.1 (54.6) ^b^	38.3 (55.2) ^b^	35.2 (55.5) ^a,b^	30.5 (59.9) ^a^	<0.001
Martin Factor	5.9 (1.5) ^a,d^	6.2 (1.8) ^b^	6.2 (1.7) ^b^	6.1 (1.7) ^a,b^	5.8 (1.5) ^c,d^	<0.001
Albumin (g/dL)	4.5 (0.4) ^a^	4.4 (0.4) ^b^	4.3 (0.3) ^b^	4.2 (0.3) ^c^	4.1 (0.4) ^d^	<0.001
Creatinine (mg/dL)	0.8 (0.1) ^a^	0.8 (0.2) ^a^	0.8 (0.2) ^a^	0.8 (0.2) ^a^	0.8 (0.2) ^b^	<0.001
eGFR (ml/min/1.73 m2)	127 (13) ^a^	118 (12) ^b^	111 (12) ^c^	102 (12) ^d^	88 (20) ^e^	<0.001

Median values and interquartile range (in parentheses) are shown. $ indicates normal distribution for such variable and age group. *Kruskal-Wallis test. a, b, c, d, e indicate different levels of significance according to Dwass-Steel-Critchlow-Fligner pairwise comparisons.

### 2.4 Pathological states assessment

We defined thresholds to distinguish regular health status against abnormal values, which could indicate pathological states (**Tables 3**, **4**). These thresholds do not diagnose disease; they identify probable pathological states that meet at least one diagnostic criterion. Overweight and obesity were considered with international cutoff points for BMI of ≥25 and ≥30, respectively. Elevated blood pressure criteria and dyslipidemia criteria were taken from (Williamson, 2025). Since eight hours of fasting was not guaranteed in all samples, the high triglycerides level cutoff was 175 mg/dL (Nordestgaard et al., 2016). Remaining cutoff points for glucose abnormalities and renal function were obtained from (American Diabetes Association, 2023) and (Análisis de sangre: la eGFR, 2021) respectively. Participants were labeled as having dyslipidemia if they met any or several of the following criteria: elevated total cholesterol (> 200 mg/dL), elevated triglycerides (> 175 mg/dL), or low levels of HDL-C (<50 mg/dL for women and <40 mg/dL for men). Dyslipidemia status is a deterministic function of three nodes of the network. Stratifying on it truncates total cholesterol, triglycerides and HDL-C in the group without dyslipidemia, which attenuates their correlations and those of LDL-C, VLDL-C, the Martin factor and METS-IR through restriction of range, and it induces negative associations among the defining variables in the group with dyslipidemia, which is selected on a disjunction of criteria (Berkson, 1946; Hernán et al., 2004). Because the prevalence of dyslipidemia varies across age strata, the intensity of this selection also varies with age, so we treat the stratified analysis as exploratory.

**Table 3:** Proportion of women that comply with criteria for different pathological states.

Women						
Pathological states	20-29 (n=971)	30-39 (n=1110)	40-49 (n=1118)	50-61 (n=998)	62-91 (n=861)	P value*
Overweight (%)	32.5	36.7	36.9	33.6	37.0	<0.001
Obesity (%)	27.8	42.3	49.4	51.7	41.1	<0.001
Hypertension (%)	11.4	20.2	34.4	47.6	57.5	<0.001
Hypercholesterolemia (%)	16.2	22.0	33.5	48.8	39.4	<0.001
Hypertriglyceridemia (%)	23.9	35.6	42.5	48.9	46.1	<0.001
Low HDL-C (%)	75.4	79.9	74.6	69.7	67.3	<0.001
Dyslipidemia (%)	83.6	88.1	88.3	89.6	86.9	<0.001
Fasting Hyperglycemia (%)	14.3	26.5	34.8	46.4	48.0	<0.001
Elevated HbA1c (%)	12.5	18.8	33.0	52.3	55.1	<0.001
Prediabetes (%)	14.3	25.2	31.8	36.2	40.4	<0.001
Diabetes (%)	4.8	8.1	14.8	27.4	27.9	<0.001
Diminished Renal Function (%)	0.3	0.5	0.5	0.5	3.9	<0.001

*Chi-squared test.

**Table 4:** Proportion of men that comply with criteria for different pathological states.

Men						
Pathological states	20-29 (n=581)	30-39 (n=518)	40-49 (n=559)	50-61 (n=605)	62-91 (n=678)	P value*
Overweight (%)	34.6	38.6	43.1	44.6	38.8	<0.001
Obesity (%)	25.1	35.1	34.3	31.9	23.5	<0.001
Hypertension (%)	30.0	41.0	46.8	53.3	56.5	<0.001
Hypercholesterolemia (%)	18.8	29.0	32.7	35.7	25.5	<0.001
Hypertriglyceridemia (%)	39.9	54.0	52.4	47.2	35.6	<0.001
Low HDL-C (%)	44.9	49.8	52.5	49.5	52.0	0.070
Dyslipidemia (%)	66.7	78.1	80.8	77.0	74.1	<0.001
Fasting Hyperglycemia (%)	15.8	21.8	32.0	44.5	40.4	<0.001
Elevated HbA1c (%)	11.9	16.8	29.5	44.6	51.3	<0.001
Prediabetes (%)	16.5	23.6	29.5	37.2	38.1	<0.001
Diabetes (%)	5.0	5.6	13.2	21.3	23.0	<0.001
Diminished Renal Function (%)	0.3	0.6	0.3	2.5	10.6	<0.001

*Chi-squared test.

### 2.5 Network construction and subsampling

The network construction methodology was based on Barajas-Martínez’s previous work (Barajas-Martínez et al., 2020). We selected Spearman’s correlation for the construction of the networks because our variables followed a non-parametric distribution according to Shapiro-Wilk tests (**Tables 1**, **2**). Significant correlations were established below a threshold of p<0.05 (**Figures 2**, **3**). Spearman’s rank coefficient was squared to obtain only positive values in our networks. Heatmap and network construction were conducted in RStudio (Posit team) using the igraph package. At a fixed significance level, the critical value of |ρ| decreases as sample size increases, so the density of a thresholded network depends on the number of observations as well as on the strength of the associations. To estimate the variability of the networks we used subsampling, a resampling scheme without replacement that is also used to assess the stability of network estimates (Epskamp et al., 2018). Subsampling was also conducted in RStudio and consisted of drawing 79% of the total sample per group without replacement and obtaining unique correlations with that data. This process was repeated 100 times for each group, and all topology measurements and network structures reflect the mean of those 100 samples. The same procedure was applied separately within the subgroups defined by age stratum, sex and the presence or absence of dyslipidemia (see Pathological States Assessment). The 95% subsampling interval (SI) of each metric was obtained from the distribution of its values across the 100 subsamples. Because subsamples of 79% share most of their observations, these intervals describe the stability of each metric under the removal of 21% of the participants and understate its sampling variability (Shao and Wu, 1989).

**Figure 2:**
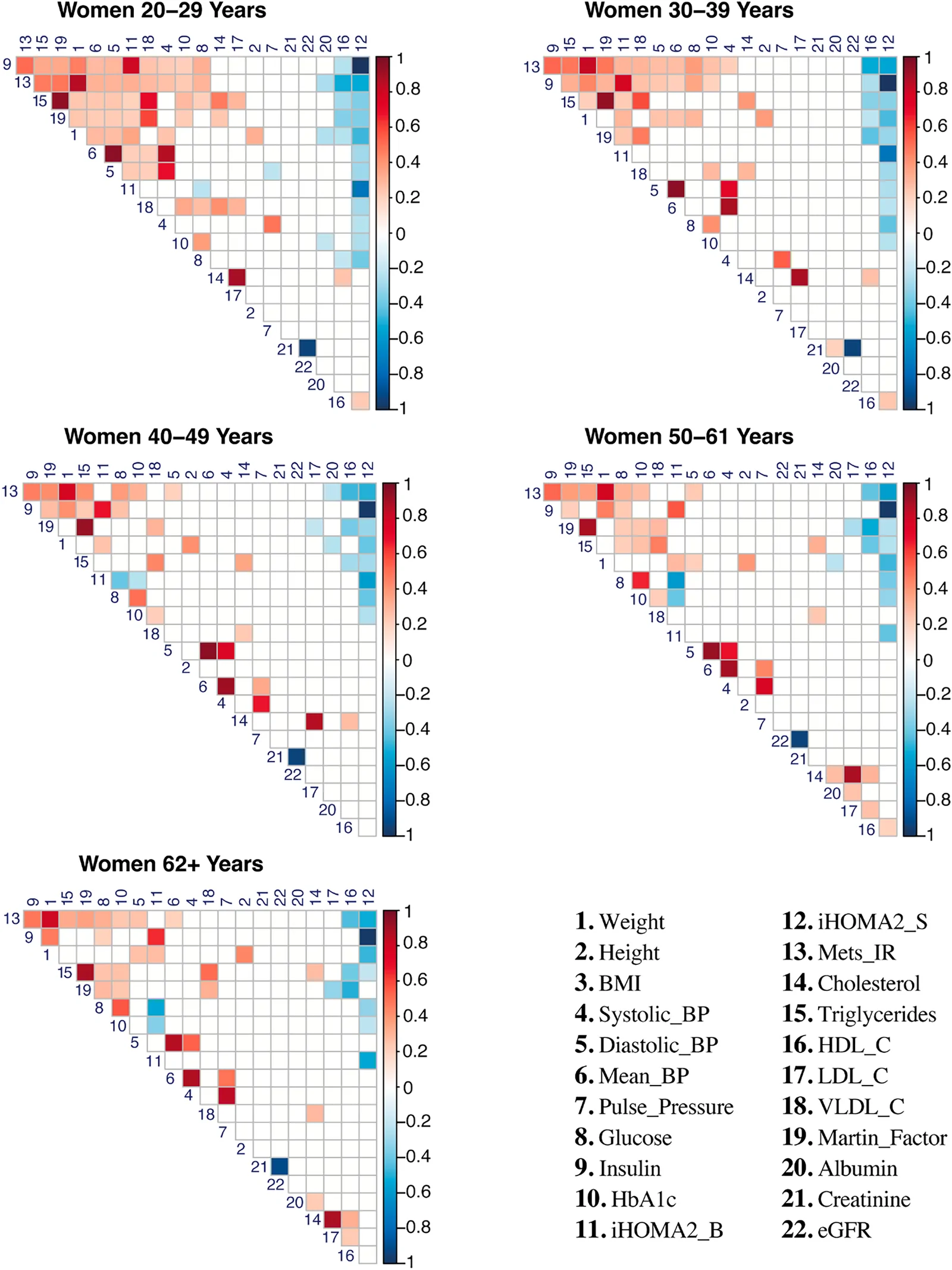
Spearman correlation heatmaps of anthropometric and biochemical variables in women. Heatmaps for correlations among all variables, sorted by First Component Analysis.

**Figure 3:**
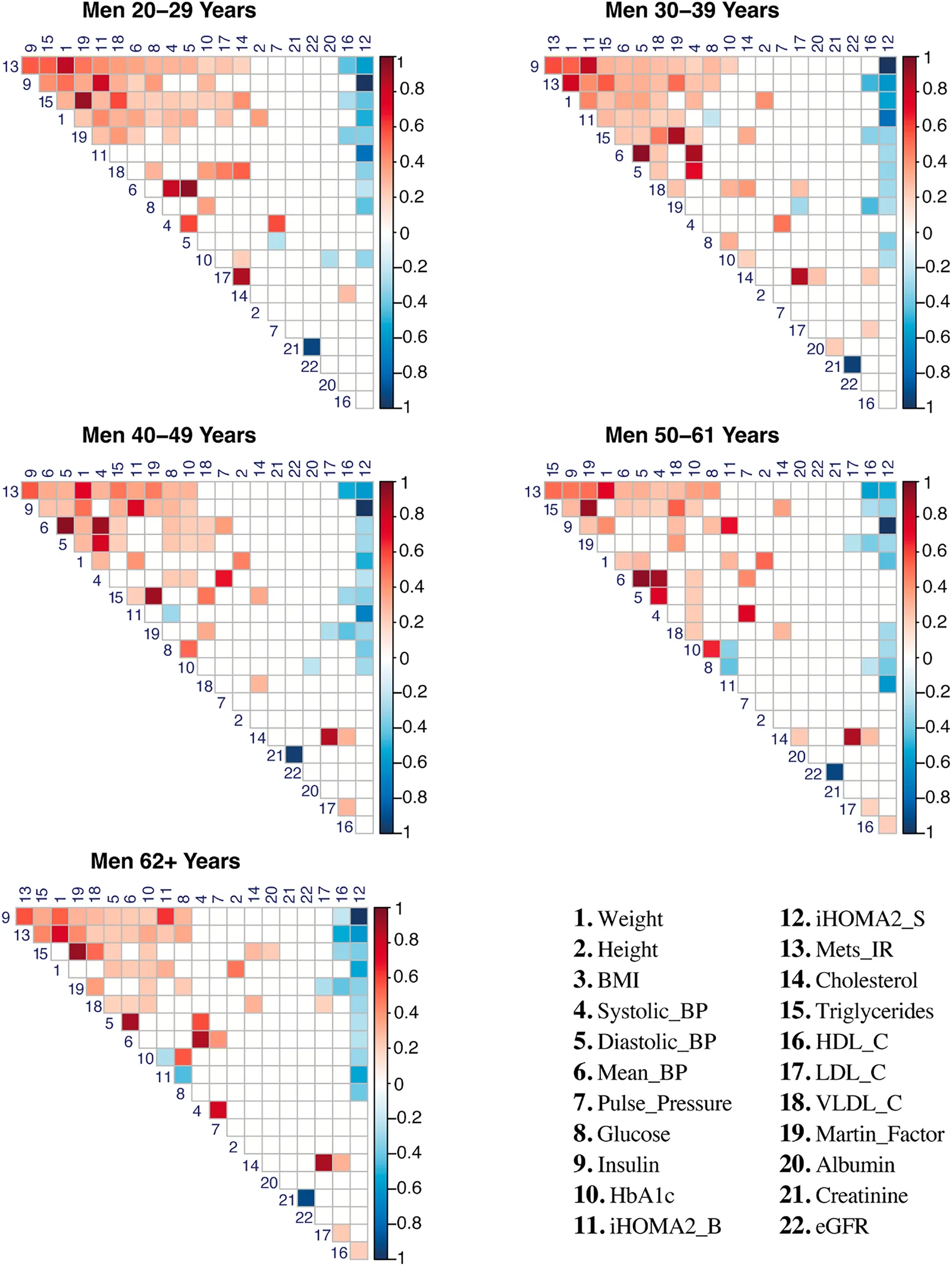
Spearman correlation heatmaps of anthropometric and biochemical variables in men. Heatmaps for correlations among all variables, sorted by First Component Analysis.

### 2.6 Network analysis

High relevance nodes in the network were assessed through degree centrality, eigen centrality and betweenness using the sna and igraph packages in RStudio. Density, transitivity, mean distance, efficiency and Louvain’s modularity were also calculated for every network with the same packages. These metrics reproduce those of our previous physiological network studies (Barajas-Martínez et al., 2020; Barajas-Martínez et al., 2021), which allows direct comparison with healthy young adults, and describe the integration, local redundancy and segregation involved in the balance between robustness and adaptability (Fossion et al., 2018; Barajas-Martínez et al., 2021). Density is the proportion of possible edges present, mean distance is the average shortest path length between connected nodes, and global efficiency is the average of the inverse shortest path lengths over all pairs of nodes (Latora and Marchiori, 2001). Transitivity is the proportion of connected triples that close into triangles, and Louvain modularity quantifies the partition of nodes that maximizes the density of within-module edges relative to a degree-preserving expectation (Blondel et al., 2008). Degree, eigenvector and betweenness centrality identify, respectively, nodes with many direct connections, nodes connected to other well-connected nodes, and nodes lying on many shortest paths between modules, and we refer to the nodes that ranked highest on these measures as hubs. Metrics that are unstable or poorly defined in networks of 22 nodes, such as the rich-club coefficient and degree assortativity, and closeness centrality, which is undefined in disconnected graphs, were not used. Density, transitivity and mean distance were computed on the unweighted graph. Global efficiency was computed on the weighted graph, where igraph interprets edge weights as lengths in path-based functions (Rubinov and Sporns, 2010), so it does not index integration in the intended sense and is reported without interpretation. Ten of the 22 variables are deterministic or near-deterministic functions of others, so part of the edges reflects construction and carries no physiological information, a problem known as mathematical coupling (Archie, 1981). We assessed its influence by removing the largest clique of each network, that is, the largest set of mutually connected nodes, and by rebuilding each network from the 12 directly measured variables alone. Edges among directly measured variables persisted in both analyses, although mathematical coupling inflates the absolute values of global metrics such as density and transitivity (see Discussion).

## 3 Results

### 3.1 Sample characteristics and univariate trajectories

The analytic sample comprised 7,999 adults, of whom 5,058 were women and 2,941 were men, distributed across five age strata (**Tables 1**, **2**). Women showed an anthropometric profile that increased until the fifth decade and then declined. Median weight moved from 63.3 kg between ages 20 and 29 to 70.3 kg between 40 and 49 and fell back to 63.0 kg in the oldest age group, while BMI followed a similar curve with a maximum of 30.2 kg/m² between ages 50 and 61. Height decreased from 156 cm to 148 cm across the strata. Hemodynamic variables rose monotonically, with systolic pressure rising from 104 to 132 mmHg and pulse pressure from 36 to 60 mmHg, the latter with the largest relative amplitude of all measured variables. Diastolic pressure peaked between the ages of 50 and 61 years and declined afterwards.

The female glycemic profile showed a dissociation between secretion and sensitivity. Fasting glucose increased from 87 to 98 mg/dL and glycated hemoglobin (HbA1c) from 5.1% to 5.8%, while insulin remained mostly unchanged across strata (p = 0.060). The iHOMA2-B index fell from 104% to 77.5%, and iHOMA2-S remained within a narrow interval between 83.7% and 94.7%; thus, glycemic deterioration was accompanied by a decline in beta-cell function with relatively preserved sensitivity. The METS-IR index grew steadily from 40.6 to 47.9 and then fell to 45.6 in the oldest group.

In contrast, female lipid profile increased through the sixth decade and declined thereafter, with total cholesterol levels ranging from 164 to 199 mg/dL, triglycerides from 120 to 173 mg/dL and LDL-C from 98 to 122 mg/dL, all peaking between the ages of 50 and 61. High-density lipoprotein cholesterol (HDL-C) varied little across strata, from 41 to 44 mg/dL. Albumin decreased from 4.3 to 4.1 g/dL, and eGFR fell from 143 to 99 mL/min/1.73 m², with creatinine levels stable at 0.6 mg/dL.

Men presented trajectories of smaller amplitudes. Weight increased from 73.9 to 77.8 kg between the third and fourth decades and fell back to 70.4 kg in the oldest group, where BMI returned to its initial value of 26.4 kg/m². Systolic blood pressure rose from 118 to 131 mmHg and pulse pressure increased from 45 to 58 mmHg. Insulin decreased from 7.9 to 6.5 µIU/mL with statistical significance, iHOMA2-B fell from 90.5% to 64.2%, and iHOMA2-S reached its highest value in the oldest group (129%). Total cholesterol and triglyceride levels peaked in the fourth and fifth decades and declined thereafter, and HDL-C was the only variable without a significant difference across strata (p = 0.27). Lastly, eGFR decreased from 127 to 88 mL/min/1.73 m².

### 3.2 Prevalence of pathological states

The proportions of participants meeting diagnostic criteria for pathological states revealed worrying trends from early ages, with a slight improvement in older age (**Tables 3**, **4**). Women had particularly high rates of dyslipidemia from an early age, mainly due to consistently low levels of HDL-C (**Table 3**). Dyslipidemia started with a prevalence of 83.6% and climbed up to 89.6% between 50 and 61 years in women. Men also showed a worsening in the prevalence of dyslipidemia, going from 66.7% between 20 and 29 years and peaking at 80.8% in the 40 to 49 years stratum. In women, hypercholesterolemia and hypertriglyceridemia increased up to the 50 to 61 years stratum and decreased slightly in the oldest group, whereas in men hypertriglyceridemia peaked between ages 30 and 39, hypercholesterolemia between 50 and 61, and both declined in the oldest group (**Tables 3**, **4**).

Obesity followed divergent trajectories between the sexes. In women, it rose from 27.8% to 51.7% with a maximum between the ages of 50 and 61, and in men, it peaked at 35.1% in the fourth decade and declined to 23.5% in the oldest group. Hypertension increased in both sexes, from 11.4% to 57.5% in women and from 30.0% to 56.5% in men, closing the nearly threefold initial gap by the seventh decade. The prevalence of diabetes increased from 4.8% to 27.9% in women and from 5.0% to 23.0% in men, with elevated glycated hemoglobin levels in more than half in both oldest groups. Reduced renal function showed the sharpest contrast, with 3.9% in women and 10.6% in men in the oldest stratum.

The low HDL-C criterion accounted for the higher prevalence of dyslipidemia in women. It was met by 67.3% to 79.9% of women, with a maximum between ages 30 and 39 and a decline thereafter, and by 44.9% to 52.5% of men, without significant variation across strata (p = 0.070). The sex-specific cutoff contributes to this difference, since median HDL-C was similar in both sexes (41–44 mg/dL in women and 39–41 mg/dL in men). Between ages 20 and 29, hypercholesterolemia differed little between sexes (16.2% in women and 18.8% in men), whereas hypertriglyceridemia was more frequent in men (39.9% versus 23.9%). Median HDL-C was lower in participants with dyslipidemia than in those without it in every stratum, with 39.8–43.0 versus 54.0–56.0 mg/dL in women and 36.0–37.0 versus 46.0–48.0 mg/dL in men (**Supplementary Tables 1**, **2**).

### 3.3 Network topology in women

Networks built from adjacency matrices derived from Spearman correlations described a two-phase trajectory in women (**Figure 4**). During the first phase, spanning the 20 to 29 stratum through the 40 to 49 stratum, density fell from 0.47 (95% SI, 0.46 – 0.47) to 0.37 (95% SI, 0.36 – 0.37) and then to 0.25 (95% SI, 0.24 – 0.25), transitivity dropped from 0.70 (95% SI, 0.70 – 0.70) to 0.63 (95% SI, 0.61 – 0.65) and to 0.50 (95% SI, 0.49 – 0.50), mean distance increased from 1.61 (95% SI, 1.61 – 1.62) to 1.90 (95% SI, 1.89 – 1.90) and to 2.32 (95% SI, 2.31 – 2.32), and Louvain modularity rose from 0.40 (95% SI, 0.40 – 0.40) to 0.45 (95% SI, 0.44 – 0.45) and to 0.56 (95% SI, 0.55 – 0.56). During the second phase, spanning ages 40 to 49 through the oldest stratum, the four metrics fluctuated within narrow bands around the values attained, with density between 0.25 (95% SI, 0.24 – 0.25) and 0.29 (95% SI, 0.28 – 0.29), transitivity between 0.50 (95% SI, 0.49 – 0.50) and 0.57 (95% SI, 0.56 – 0.57), mean distance between 2.17 (95% SI, 2.16 – 2.17) and 2.32 (95% SI, 2.31 – 2.32), and modularity between 0.55 (95% SI, 0.54 – 0.55) and 0.56 (95% SI, 0.56 – 0.56). The 40 to 49 stratum recorded the most extreme value of all four metrics, and the subsequent variation of each was smaller than that accumulated up to that point.

**Figure 4:**
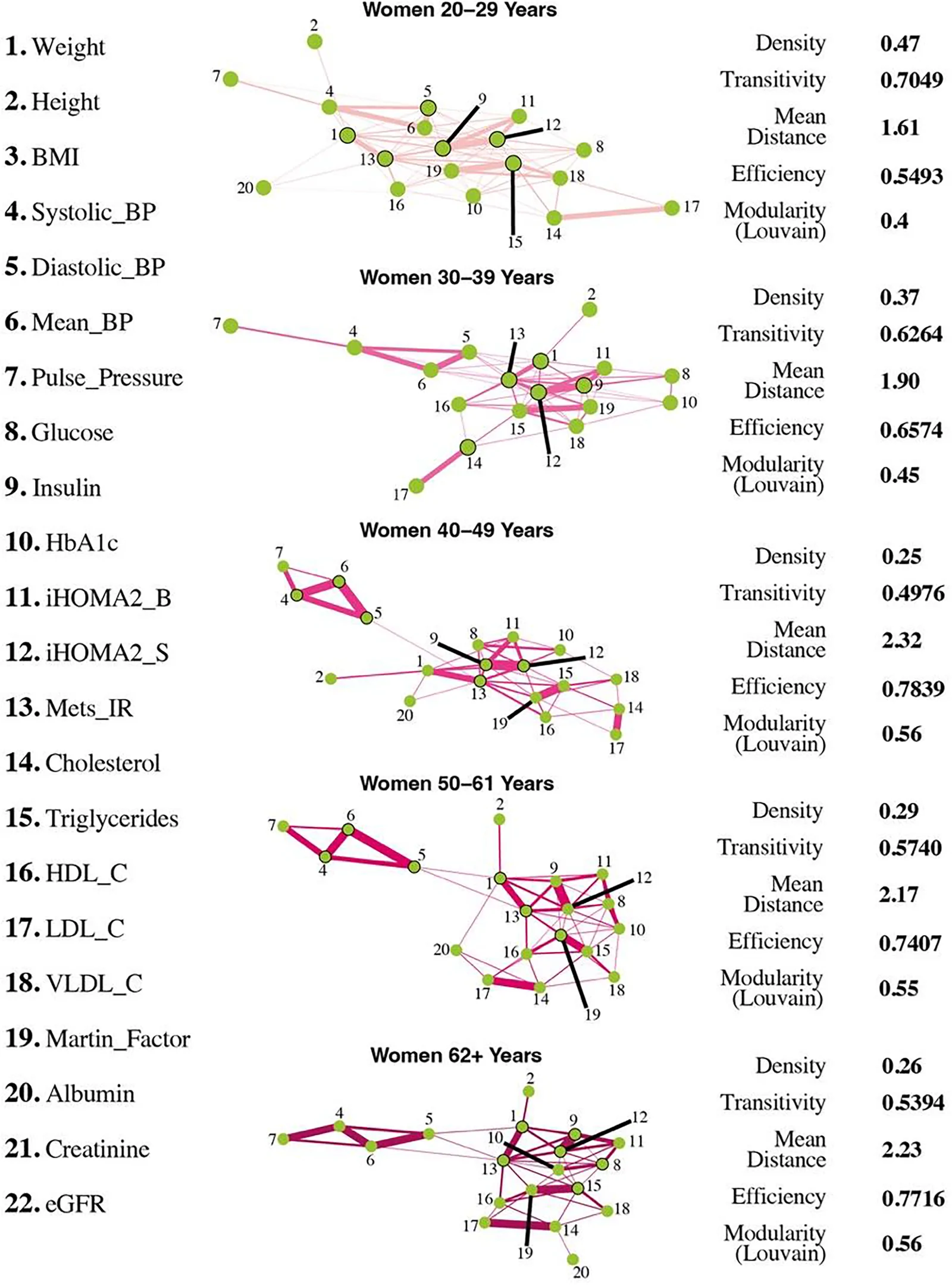
Changes in network topology in women throughout the years. Undirected, weighted networks built from adjacency matrices derived from Spearman correlations. Only the largest connected component was analyzed and shown. Black contouring shows the nodes with most degree centrality, eigen centrality and betweenness. Thickness of the edge is proportional to strength of the square of the correlation. On the right, tables describing main topology variables from each network.

Efficiency, computed on the weighted graph, followed the same biphasic form in an ascending direction, from 0.55 (95% SI, 0.53 – 0.56) to 0.66 (95% SI, 0.65 – 0.66) and to 0.78 (95% SI, 0.77 – 0.78) during the first phase, with subsequent fluctuations between 0.74 (95% SI, 0.73 – 0.74) and 0.78 (95% SI, 0.77 – 0.78). Because igraph interprets edge weights as lengths in path-based functions, weighted efficiency does not index integration in the intended sense, and we report it without interpreting it (see Methods). Density, transitivity, mean distance and modularity describe a single process, in which the female network loses edges, loses triangles, lengthens its paths and becomes compartmentalized.

The univariate trajectories described in Section 3.1 varied gradually across the five strata, without a comparable discontinuity in the window from 30 to 49 years. The topological series therefore locates a discontinuity that the marginal series does not show, and the values reached after it hold for two further decades within the resolution of these metrics.

Further inspection of the networks identified the structural pattern of the transition. Between the ages of 20 and 29, the hemodynamic variables appeared integrated into the main body of the network, with direct edges among mean pressure, weight, METS-IR and insulin. From age 40 onward, the set formed by systolic, diastolic, mean, and pulse blood pressure became separated as a differentiated module, joined to the glycemic and lipid core by a reduced number of edges. The nodes with the highest degree centrality, eigenvector centrality, and betweenness consistently corresponded to METS-IR, insulin, weight and the iHOMA2 indices, with the recurrent presence of directly measured variables such as weight and systolic pressure among the outlined nodes.

Creatinine and eGFR remained outside the largest connected component in all five female strata and formed a two-node island joined by construction, explained by the formula that estimates eGFR from the value of creatinine.

### 3.4 Network topology in men

Male networks did not exhibit a comparable trajectory (**Figures 5**, **6**). Density moved within the band from 0.34 to 0.42 with values of 0.42 (95% SI, 0.41 – 0.42), 0.34 (95% SI, 0.33 – 0.34), 0.38 (95% SI, 0.37 – 0.38), 0.35 (95% SI, 0.34 – 0.35), and 0.38 (95% SI, 0.37 – 0.38) in ascending order of age, transitivity within the band from 0.56 (95% SI, 0.55 – 0.56) to 0.69 (95% SI, 0.68 – 0.69), mean distance between 1.72 (95% SI, 1.71 – 1.72) and 2.19 (95% SI, 2.18 – 2.19), and efficiency between 0.61 (95% SI, 0.60 – 0.61) and 0.69 (95% SI, 0.68 – 0.69). The only directional variation corresponded to modularity, which rose from 0.37 (95% SI, 0.36 – 0.37) to 0.45 (95% SI, 0.44 – 0.45) between the third and fourth decades and remained between 0.44 (95% SI, 0.43 – 0.44) and 0.47 (95% SI, 0.46 – 0.47) thereafter. The range of male density across all strata (0.34 to 0.42) was smaller than the change in female density between two consecutive strata (0.47 to 0.37 and 0.37 to 0.25).

**Figure 5:**
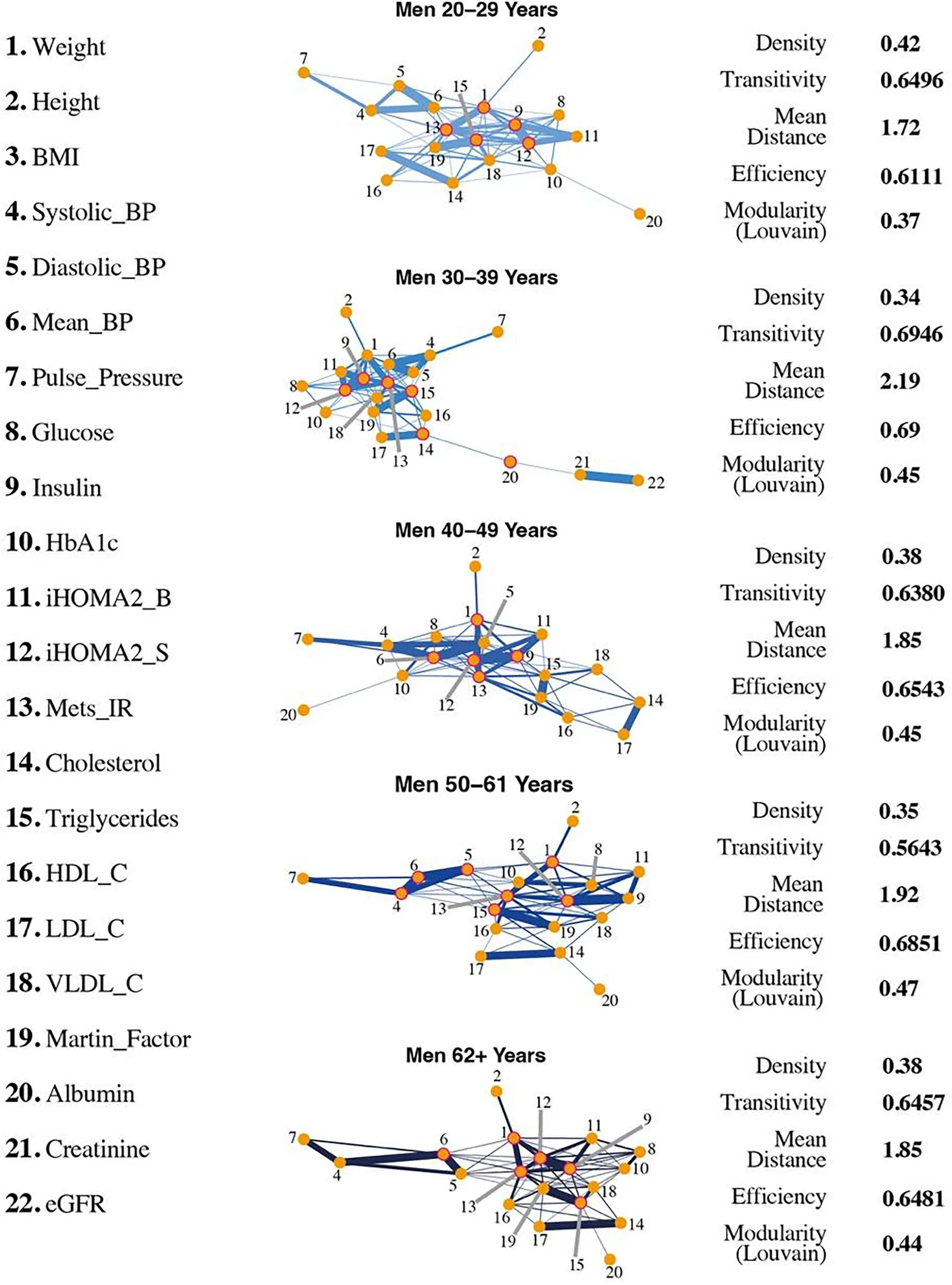
Changes in network topology in men throughout the years. Undirected, weighted networks built from adjacency matrices derived from Spearman correlations. Only the largest connected component was analyzed and shown. Pink contouring shows the nodes with most degree centrality, eigen centrality and betweenness. Thickness of the edge is proportional to strength of the square of the correlation. On the right, tables describing main topology variables from each network.

**Figure 6:**
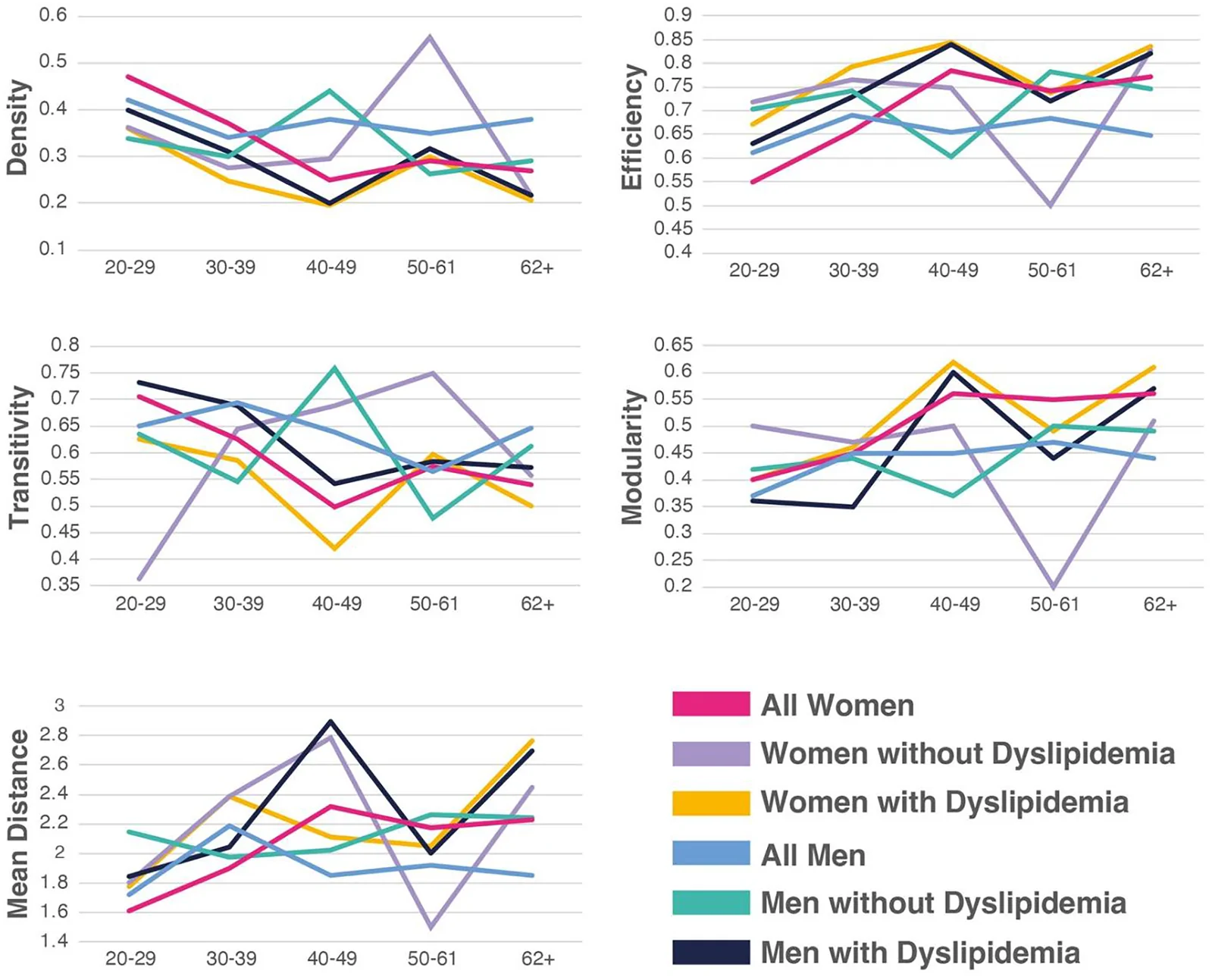
Changes in network topology according to age and dyslipidemia.

The initial modularity step is worth recording because both sexes share it. Between the third and fourth decades, the values for women rose from 0.40 to 0.45 and for men from 0.37 to 0.45, magnitudes that are almost identical. From that point, the trajectories separated, since women continued to 0.56, while men stopped. The between-sex comparison further showed that men started from a less dense and less modular configuration than women and ended in a denser and less modular configuration (density 0.38 versus 0.26; modularity 0.44 versus 0.56), with the density trajectories crossing between the fourth and fifth decades.

### 3.5 Effect of dyslipidemia on topology

In women, networks of the subgroup with dyslipidemia followed the age trajectory of the full female sample in density and modularity, with lower density at most strata (**Figures 7**, **6**). Networks of women without dyslipidemia fluctuated without a consistent age pattern and reached their highest density and lowest modularity between ages 50 and 61. Because dyslipidemia status conditions on three nodes of the network and the subgroups differ in size, these differences are not interpreted as effects of dyslipidemia. Other than calculated variables such as METS-IR, BMI and iHOMA2-S, weight and insulin were consistently nodes of high relevance in female networks with or without dyslipidemia (**Figure 7**).

**Figure 7:**
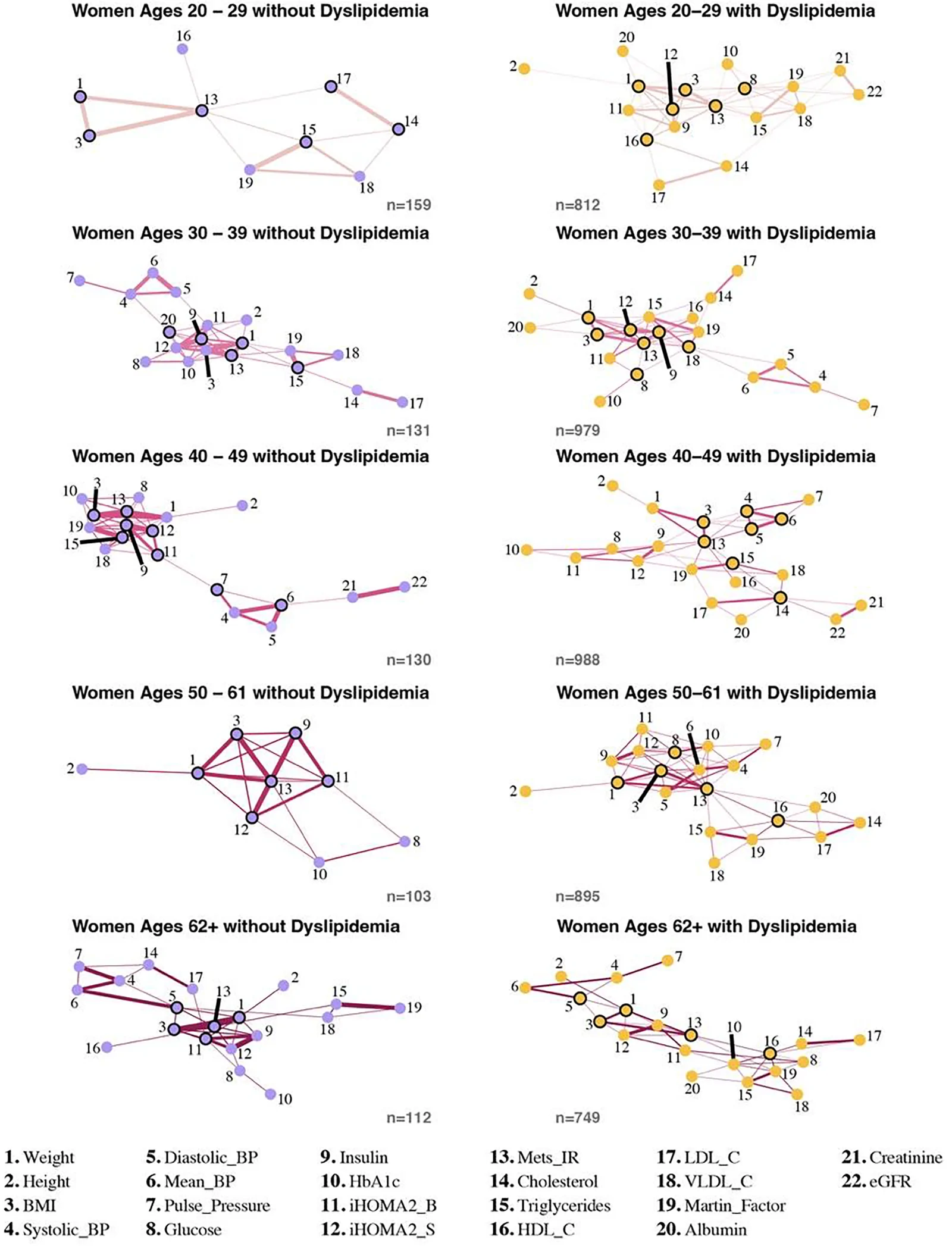
Physiological networks of women with and without dyslipidemia. Undirected, weighted networks built from adjacency matrices derived from Spearman correlations. Only the largest connected component was analyzed and shown. Black contouring shows the nodes with most degree centrality, eigen centrality and betweenness. Thickness of the edge is proportional to strength of the square of the correlation.

As for men (**Figure 8**), nodes of high relevance were essentially the same as their female counterparts, with or without dyslipidemia. Men without dyslipidemia exhibited slight changes in all topology metrics from the youngest to the oldest age, whereas the middle strata fluctuated considerably. Men with dyslipidemia exhibited wider variations in topology, with minima of density and maxima of mean distance at ages 40 to 49 and 62 and older (**Figure 6**).

**Figure 8:**
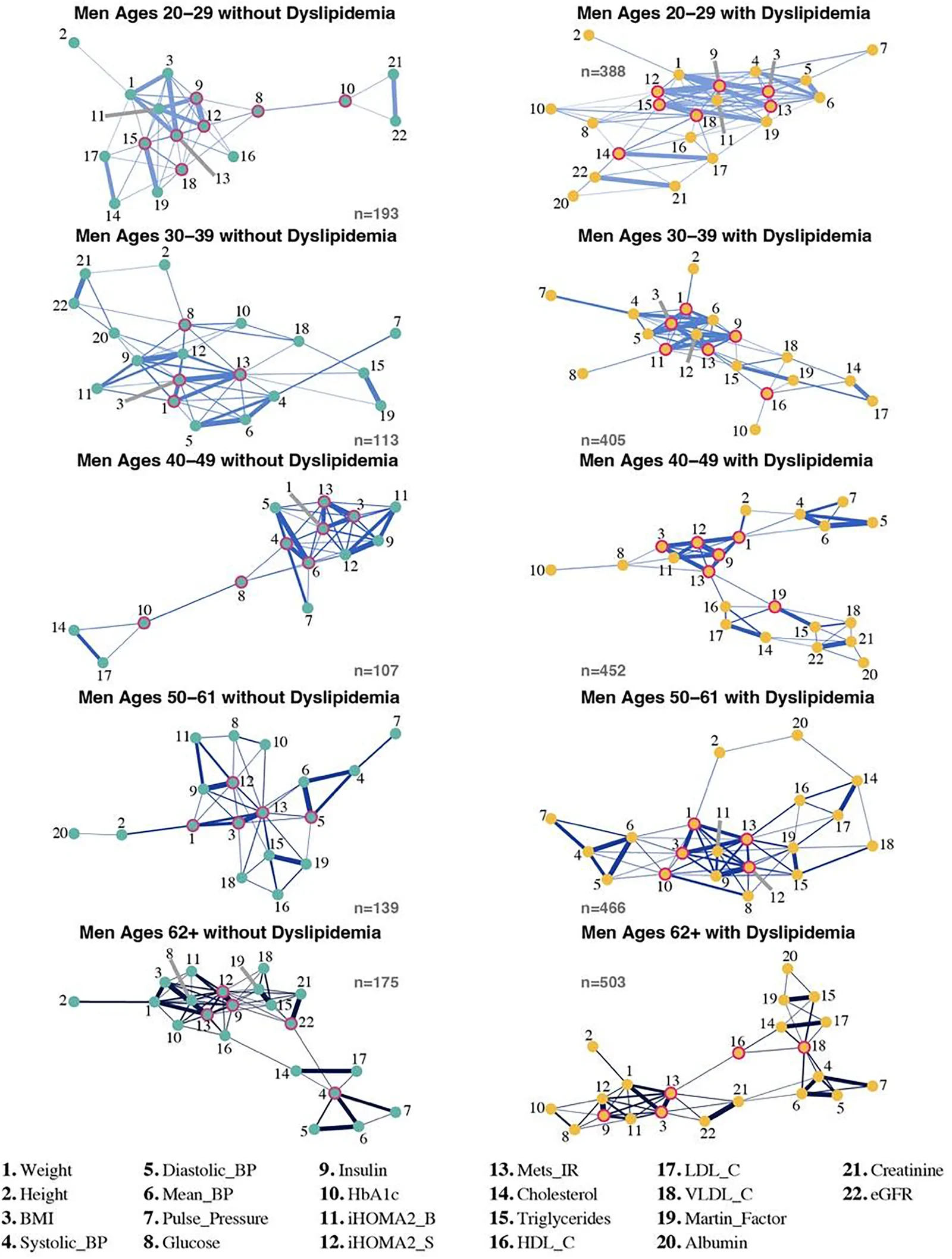
Physiological networks of men with and without dyslipidemia. Undirected, weighted networks built from adjacency matrices derived from Spearman correlations. Only the largest connected component was analyzed and shown. Pink contouring shows the nodes with most degree centrality, eigen centrality and betweenness. Thickness of the edge is proportional to strength of the square of the correlation.

Overall, the networks tended toward sparser configurations with age in both subgroups, and dyslipidemia did not fully account for these transitions. Comparisons between subgroups are limited by their unequal sizes and by the conditioning on nodes of the network described in the Methods, so these results are exploratory.

## 4 Discussion

Unlike women, men do not exhibit drastic changes in network topology throughout life. This absence could be explained by the floor effect, according to which the transition women undergo during the fifth decade occurs in men before age 20. This explanation could be directly tested in cohorts aged 12–20 years, where the male transition should be observable. An alternative would be to consider men as physiologically stable throughout their lifetime; however, this hypothesis may be greatly skewed by survivor bias. Men exhibit higher premature mortality from cardiovascular disease, diabetes, and renal disease from midlife onward (Gómez-Dantés et al., 2016), so that each successive male stratum in our study represents an increasingly selected fraction of its cohort, purged of the worst cardiometabolic profiles.

Homeostasis is maintained by effector variables that return controlled variables to a region of relative stability (Fossion et al., 2018). The organism is not perfectly elastic: damage accumulates, physiological reserve declines, and effectors whose response range narrows can no longer dampen fluctuations in the variables they regulate. Because the networks are built from correlations across individuals, a narrowing of effector ranges shared by a population would alter the covariation among variables even when their marginal distributions change little. This is what these series show: the univariate trajectories of Section 3.1 shift gradually in both sexes, while the topological trajectories separate sharply by sex.

The networks describe how physiological variables covary across the individuals of each age–sex stratum. An edge indicates that individuals who rank high on one variable tend to rank high, or low, on another, and it does not establish that the two variables are coupled within any given person. Between-person structure matches the regulatory structure of individuals only when individuals are homogeneous and their dynamics stationary, conditions that cross-sectional data cannot test (Molenaar, 2004; Fisher et al., 2018). The loss of density and the rise in modularity therefore describe a reorganization of inter-individual variability, and the reading in terms of effector ranges proposed above remains a hypothesis about individual physiology. Repeated measurements within persons, such as the continuous monitoring described by Fossion et al. (2018), would test it by estimating individual networks and comparing their topology with the population networks reported here.

In women, the young network is dense and highly transitive, a configuration with redundant paths between biomarkers; both properties decline while modularity rises, so the older female network is more compartmentalized and retains fewer alternative routes between the same variables.

This reorganization concentrates between ages 30 and 49 and then holds. The median age at menopause in Mexican women is approximately 48 years (Legorreta et al., 2013), and the menopausal transition begins several years earlier (El Khoudary et al., 2020), so the 40 to 49 stratum overlaps it. The decline in density and transitivity was already present between the 20 to 29 and 30 to 39 strata, before the menopausal transition, so the reorganization cannot be attributed to menopause alone, even though menopause is accompanied by changes in lipid regulation and vascular reorganization (Matthews et al., 2009; El Khoudary et al., 2020). Allostatic load appears to act on the architecture from early adulthood onward.

Stratification by dyslipidemia follows the general trends of aging, with variations across the middle strata that show no clear pattern. Because dyslipidemia status is defined by three nodes of the network and the subgroups differ in size, these variations are compatible with selection and sampling effects and are not discussed as effects of the disease.

Retaining edges at a fixed significance level makes network density depend on sample size. Within each sex, stratum sizes varied little, and the largest decline in female density occurred while stratum size increased from 971 to 1,118 participants, the opposite of what a sample-size artifact would produce. Female strata were 1.3 to 2.1 times larger than male strata, which favors denser female networks, yet the female networks were sparser than the male ones from age 40 onward. The higher density of the two youngest female strata, and every comparison between dyslipidemia subgroups, remain compatible with an effect of sample size. The subsampling intervals understate sampling variability because subsamples overlap (Shao and Wu, 1989), but doubling their width, a correction larger than the one the delete-d jackknife gives for this overlap, leaves the intervals of consecutive female strata separated during the first phase for density, transitivity, mean distance and modularity.

This study presents key limitations that bound the scope of its conclusions: the cross-sectional design confounds age, cohort and survival effects; the use of Spearman correlations prevents distinguishing direct from indirect associations; and the inclusion of calculated variables inflates global metrics such as density and transitivity, although two controls (the subnetwork without calculated variables and removal of the maximal *clique*) retained edges among directly measured variables. Network stability is likewise affected by sample size in reduced subgroups, compounded by the exclusion of nodes outside the largest connected component, and the absence of key factors in life such as inflammatory markers, neuroendocrine measures and other lifestyle factors such as dieting and exercise. Further constraints bound the reading of the networks. Survey weights were not applied, so the estimates describe the participants sampled and are not generalizable to the Mexican population. Edges were retained at p < 0.05 without correction for multiplicity, and each network involves 231 simultaneous tests, so a fraction of the retained edges is expected to be spurious. Squaring Spearman’s coefficient discards the sign of each association, so the network metrics do not distinguish direct from inverse coupling. Pooling three surveys can create covariation that exists in none of them when two variables shift in the same direction between surveys, a possibility that the present analysis did not examine.

## 5 Conclusion

Physiological networks in women show high connectivity in early adulthood, with a reorganization concentrated between the ages of 30 and 49 years and a subsequent plateau, during which the system loses density and redundancy, and converges toward the modular configuration that characterizes healthy young adult men. Men preserve an established architecture across the age range examined, without a comparable reorganization accompanying the rise in disease prevalence and the shifts in biochemical markers observed over the same strata. These networks describe the covariation of allostatic load biomarkers across individuals, and their reading as regulatory architecture within persons requires longitudinal confirmation. The stratified analysis, although exploratory, suggests that dyslipidemia does not explain by itself the changes in network topology during aging, and edges among directly measured variables indicate that connectivity is not attributable to the derived variables alone. These results extend to adult life the sexual dimorphism previously described in the physiological networks of healthy young adults.

## Data Availability

Publicly available datasets were analyzed in this study. This data can be found here: This study is a secondary analysis of publicly available data retrieved from the National Health and Nutrition Survey (ENSANUT) repository (https://ensanut.insp.mx/). The original survey protocols, sampling methodology, and consent procedures were reviewed and approved by the Ethics, Research, and Biosafety Committees of the National Institute of Public Health (Instituto Nacional de Salud Pública, INSP) of Mexico. Supplementary tables can be consulted in the following link: Sampieri-Cabrera, R. (2026). table_. Zenodo. https://doi.org/10.5281/zenodo.23110605.
