# Supplementary material for: Topological transition at midlife in the physiological network of Mexican women and divergent amplitude of topological change between sexes: allostatic load biomarkers across adult life
Source: Front Physiol. 2026 Oct 21;17:1982421. doi: 10.3389/fphys.2026.1982421 (PMC13730928; doi:10.3389/fphys.2026.1982421)
Supplement: Supplementary file 1 [file Table1.docx]

| **Women** | | | | | | | | | | | |
| --- | --- | --- | --- | --- | --- | --- | --- | --- | --- | --- | --- |
|  | **Without Dyslipidemia** | | | | | **With Dyslipidemia** | | | | |  |
|  | **20-29**  **(n =159)** | **30-39**  **(n =131)** | **40-49**  **(n =130)** | **50-61**  **(n =103)** | **62+**  **(n =112)** | **20-29**  **(n =812)** | **30-39**  **(n =979)** | **40-49**  **(n =988)** | **50-61**  **(n =895)** | **62+**  **(n =749)** | **P Value*** |
| **Weight (kg)** | 58.0 (16.3) $ | 63.1 (16.0) $ | 64.4 (16.8) $ | 70.1 (19.5) | 61.9 (17.4) | 64.7 (19.4) $ | 68.8 (18.2) $ | 70.8 (17.7) $ | 69.4 (18.4) | 63.3 (17.1) | <0.001 |
| **Height (cm)** | 157 (6.8) | 154 (7.7) | 154 (8.2) | 152 (10.0) | 147 (11.5) | 155 (9.0) | 154 (8.5) | 153 (8.9) | 152 (8.3) | 149 (8.1) | <0.001 |
| **BMI (Kg/m^2^)** | 24.0 (6.0)$ | 26.5 (7.4) $ | 26.8 (5.4) $ | 30.7 (7.0) | 29.2 (6.7) | 27.0 (7.4) $ | 29.2 (7.0) $ | 30.3 (6.7) $ | 30.1 (7.1) | 28.6 (6.6) | <0.001 |
| **Systolic Blood Pressure (mmHg)** | 101 (13.6) | 106 (16.1) | 115 (21.3) | 121 (26.0) | 131 (23.8) | 105 (13.2) | 109 15.5() | 115 (20.5) | 124 (23.3) | 133 (29.5) | <0.001 |
| **Diastolic Blood Pressure (mmHg)** | 66 (11.5) | 69 (12.8) | 72 (16.0) | 74 (15.7) | 70 (13.9) | 68 (12.0) | 71 (12.0) | 74 (14.6) | 75 (14.7) | 72 (15.0) | <0.001 |
| **Mean Blood Pressure (mmHg)** | 78 (10.8) | 82 (12.7) | 86 (18.1) | 90 (18.0) | 91 (15.8) | 80 (11.6) | 83 (12.3) | 87 (15.5) | 91 (16.3) | 92 (18.2) | <0.001 |
| **Pulse Blood Pressure (mmHg)** | 35 (9.0) | 34 (10.8) | 40 (14.7) | 45 (14.0) | 58 (21.2) | 36 (9.3) | 37 (10.8) | 40 (12.7) | 47 (16.0) | 60 (24.5) | <0.001 |
| **Glucose (mg/dL)** | 84 (8.5) $ | 89 (13.0) $ | 91 (15.8) $ | 94 (16.8) | 93 (14.2) $ | 88 (13.8) $ | 93 (13.3) $ | 95 (19.0) $ | 98 (33.0) | 100 (31.5) $ | <0.001 |
| **Insulin (μUI/mL)** | 7.2 (5.8) $ | 7.1 (6.5) $ | 7.7 (6.0) $ | 8.4 (8.8) | 8.7 (7.8) | 10.2 (9.8) $ | 10.6 (9.6) $ | 10.4 (8.9) $ | 10.  5 (9.5) | 9.9 (9.2) | <0.001 |
| **HbA1c (%)** | 5.1 (0.4) $ | 5.2 (0.4) $ | 5.4 (0.5) | 5.7 (0.7) | 5.5 (0.6) $ | 5.2 (0.5) $ | 5.3 (0.6) $ | 5.5 (0.6) | 5.7 (1.1) | 5.8 (1.1) $ | <0.001 |
| **iHOMA2-B (%)** | 90.5 (48.1) | 86.0 (44.2) | 84.2 (44.3) | 83.0 (56.4) | 81.1 (46.8) | 108.0 (63.7) | 101.0 (57.8) | 92.9 (56.2) | 84.0 (58.1) | 76.2 (58.3) | <0.001 |
| **iHOMA2-S (%)** | 129.0 (98.3) $ | 131.0 (110.0) $ | 110.0 (82.6) $ | 99.9 (100.0) | 101.0 (103) | 89.2 (82.0) $ | 84.3 (72.2) $ | 84.5 (67.4) $ | 82.2 (71.5) | 87.0 (80.6) | <0.001 |
| **METS-IR** | 32.9 (9.1) $ | 37.6 (11.3) $ | 38.6 (9.2) $ | 43.5 (10.0) $ | 41.6 (11.1) $ | 42.3 (14.6) $ | 47.2 (13.7) $ | 49.0 (12.6) $ | 48.7 (14.1) $ | 46.2 (13.3) $ | <0.001 |
| **Cholesterol (mg/dL)** | 164 (31.0) | 171 (22.5) | 179 (27.8) $ | 179 (25.0) $ | 180 (22.0) $ | 164 (47.0) | 173 (48.0) | 189 (54.0) $ | 204 (55.0) $ | 196 (53.3) $ | <0.001 |
| **Triglycerides (mg/dL)** | 82 (45.0) $ | 91 (51.0) $ | 94 (46.8) $ | 106 (44.0) $ | 114 (48.0) $ | 131 (92.0) $ | 157 (102.0) $ | 171 (108.0) $ | 183 (108.0) $ | 181 (106.0) $ | <0.001 |
| **HDL-C (mg/dL)** | 56.0 (9.5) $ | 55.4 (7.0) $ | 56.0 (9.6) $ | 54.0 (8.1) $ | 56.0 (8.2) $ | 41.0 (11.0) $ | 39.8 (11.0) $ | 41.8 (11.0) $ | 43.0 (11.8) $ | 42.8 (12.0) $ | <0.001 |
| **LDL-C (mg/dL)** | 93.0 (28.0) $ | 96.7 (24.4) $ | 104.0 (22.8) $ | 104.0 (23.7) $ | 101.0 (19.3) $ | 99.7 (37.6) $ | 105.0 (35.2) $ | 115.0 (39.5) $ | 126.0 (43.0) $ | 118.0 (43.9) $ | <0.001 |
| **VLDL-C (mg/dL)** | 14.0 (26.6) $ | 17.6 (56.0) $ | 21.1 (77.8) $ | 24.8 (82.9) | 22.5 (74.6) $ | 24.4 (41.0) $ | 28.7 (32.6) $ | 33.7 (50.9) $ | 37.3 (56.3) | 35.1 (50.7) $ | <0.001 |
| **Martin Factor** | 4.8 (1.0) $ | 5.1 (0.9) $ | 5.1 (0.8) $ | 5.2 (0.9) $ | 5.4 (0.8) $ | 5.7 (1.5) $ | 6.0 (1.4) $ | 6.2 (1.5) $ | 6.0 (1.4) $ | 6.0 (1.5) $ | <0.001 |
| **Albumin (g/dL)** | 4.4 (0.4) $ | 4.2 (0.3) | 4.1 (0.4) | 4.1 (0.4) | 4.1 (0.4) | 4.2 (0.4) $ | 4.2 (0.  4) | 4.1 (0.4) | 4.2 (0.4) | 4.1 (0.4) | <0.001 |
| **Creatinine (mg/dL)** | 0.6 (0.1) | 0.6 (0.1) | 0.6 (0.1) | 0.6 (0.1) | 0.7 (0.1) | 0.6 (0.1) | 0.6 (0.1) | 0.6 (0.1) | 0.6 (0.1) | 0.6 (0.2) | <0.001 |
| **eGFR (ml/min/1.73 m2)** | 144 (14.3) | 132 (13.4) | 125 (12.1) | 115 (11.7) | 97 (14.0) | 143 (14.6) | 132 (13.2) | 124 (13.7) | 112 (13.3) | 99 (15.2) | <0.001 |

**Supplementary Table 1 (S1). Descriptive Statistics of Women with and without Dyslipidemia.** Median values and interquartile range (in parentheses) are shown. $ indicates significant difference compared to the same age group according to Dwass-Steel-Critchlow-Fligner pairwise comparisons.* Kruskal-Wallis test.
