# Supplementary material for: Topological transition at midlife in the physiological network of Mexican women and divergent amplitude of topological change between sexes: allostatic load biomarkers across adult life
Source: Front Physiol. 2026 Oct 21;17:1982421. doi: 10.3389/fphys.2026.1982421 (PMC13730928; doi:10.3389/fphys.2026.1982421)
Supplement: Supplementary file 2 [file Table2.docx]

| **Men** | | | | | | | | | | | |
| --- | --- | --- | --- | --- | --- | --- | --- | --- | --- | --- | --- |
|  | **Without Dyslipidemia** | | | | | **With Dyslipidemia** | | | | |  |
|  | **20-29**  **(n =193)** | **30-39**  **(n =113)** | **40-49**  **(n =107)** | **50-61**  **(n =139)** | **62+**  **(n =175)** | **20-29**  **(n =388)** | **30-39**  **(n =405)** | **40-49**  **(n =452)** | **50-61**  **(n =466)** | **62+**  **(n =503)** | **P Value*** |
| **Weight (kg)** | 69.1 (17.8) $ | 72.7 (18.1) $ | 72.7 (22.6) $ | 73.8 (17.9) | 65.4 (18.2) $ | 77.2 (23.4) $ | 79.3 (20.9) $ | 78.5 (18.6) $ | 77.3 (17.6) | 71.7 (16.8) $ | <0.001 |
| **Height (cm)** | 170 (9.7) | 169 (10.3) | 166 (10.7) | 166 (10.4) | 162 (10.8) | 168 9.5() | 166 (8.6) | 166 (9.3) | 164 (9.1) | 162 (8.9) | <0.001 |
| **BMI (Kg/m^2^)** | 24.3 (6.2) $ | 25.5 (6.7) $ | 26.0 (6.8) $ | 27.0 (5.0) $ | 25.2 (5.7) $ | 27.3 (7.3) $ | 28.6 (6.5) $ | 28.4 (5.4) $ | 28.4 (5.7) $ | 26.8 (5.9) $ | <0.001 |
| **Systolic Blood Pressure (mmHg)** | 118 (18.7) | 119 (11.7) | 122 (18.6) | 125 (19.6) | 129 (26.5) | 119 (17.1) | 122 (16.4) | 122 (19.5) | 127 (20.9) | 133 (24.5) | <0.001 |
| **Diastolic Blood Pressure (mmHg)** | 71 (13.7) | 74 (12.3) | 76 (15.0) | 77 (14.9) | 71 (15.2) | 72 (14.3) | 76 (14.6) | 77 (13.9) | 78 (14.0) | 73 (15.2) | <0.001 |
| **Mean Blood Pressure (mmHg)** | 87 (13.0) | 88 (10.3) | 91 (15.2) | 92 (15.7) | 89 (20.4) | 88 (13.5) | 92 (14.6) | 92 (16.2) | 94 (15.6) | 92 (16.7) | <0.001 |
| **Pulse Blood Pressure (mmHg)** | 44 (12.7) | 45 (8.3) | 44 (13.2) | 48 (12.8) | 55 (21.4) | 45 (11.8) | 44 (11.7) | 46 (12.2) | 49 (14.0) | 58 (19.5) | <0.001 |
| **Glucose (mg/dL)** | 87 (10.2) | 90 (13.7) | 91 (13.8) | 94 (15.5) $ | 93 (17.0) | 89 (13.0) | 91 (12.0) | 94 (16.6) | 99 (28.5) $ | 96 (24.5) | <0.001 |
| **Insulin (μUI/mL)** | 6.1 (5.3) $ | 5.8 (5.9) $ | 6.1 (7.2) $ | 6.9 (7.2) $ | 5.2 (5.5) $ | 9.1 (9.3) $ | 8.9 (9.4) $ | 8.8 (8.9) $ | 8.4 (9.6) $ | 7.2 (7.3) $ | <0.001 |
| **HbA1c (%)** | 5.1 (0.5) | 5.3 (0.5) | 5.3 (0.3) | 5.5 (0.5) $ | 5.5 (0.7) $ | 5.2 (0.4) | 5.3 (0.4) | 5.5 (0.6) | 5.6 (0.7) $ | 5.7 (0.9) $ | <0.001 |
| **iHOMA2-B (%)** | 77.8 (41.5) $ | 72.2 (42.7) $ | 74.2 (58.8) | 67.9 (56.9) | 57.8 (41.7) | 97.6 (62.8) $ | 91.9 (61.5) $ | 84.7 (60.5) | 71.3 (56.4) | 66.0 (53.4) | <0.001 |
| **iHOMA2-S (%)** | 148.0 (120.0) $ | 152.0 (143.0) $ | 142.0 (167.0) $ | 122.0 (134.0) $ | 166.0 (149.0) $ | 98.9 (102.0) $ | 99.7 (92.2) $ | 99.9 (96.1) $ | 99.4 (105.0) $ | 119.0 (115.0) $ | <0.001 |
| **METS IR** | 35.5 (10.5) $ | 37.5 (9.2) $ | 37.6 (10.9) $ | 40.5 (9.0) $ | 37.4 (9.4) $ | 44.9 (13.9) $ | 47.8 (13.1) $ | 48.1 (12.3) $ | 47.6 (13.0) $ | 45.4 (11.2) $ | <0.001 |
| **Cholesterol (mg/dL)** | 165 (37.0) $ | 171 (26.0) $ | 176 (25.5) $ | 169 (31.5) $ | 167 (30.0) $ | 174 (53.0) $ | 185 (55.0) $ | 190 (54.3) $ | 196 (50.0) $ | 182 (53.5) $ | <0.001 |
| **Triglycerides (mg/dL)** | 100 (52.0) $ | 106 (54.0) $ | 108 (42.0) $ | 112 (56.5) $ | 110 (47.5) $ | 187 (116.0) $ | 212 (128.0) $ | 212 (144.0) $ | 200 (138.0) $ | 169 (123.0) $ | <0.001 |
| **HDL-C (mg/dL)** | 48.0 (12.0) $ | 47.1 (10.9) $ | 48.0 (11.0) $ | 46.0 (8.4) $ | 46.0 (9.8) $ | 36.7 (10.5) $ | 37.0 (11.1) $ | 37.0 (11.0) $ | 37.0 (11.0) $ | 36.0 (9.0) $ | <0.001 |
| **LDL-C (mg/dL)** | 97.0 (33.0) $ | 102.0 (29.6) $ | 104.0 (25.0) $ | 103.0 (29.7) $ | 101.0 (29.0) $ | 104.0 (40.1) $ | 113.0 (45.9) $ | 113.0 (43.5) $ | 118.0 (40.9) $ | 110.0 (42.8) $ | <0.001 |
| **VLDL-C (mg/dL)** | 20.1 (69.6) $ | 22.5 (77.2) $ | 19.9 (63.7) $ | 22.8 (68.5) $ | 20.7 (70.7) $ | 35.4 (56.6) $ | 38.2 (42.8) $ | 40.8 (51.1) $ | 38.3 (48.4) $ | 33.5 (53.9) $ | <0.001 |
| **Martin Factor** | 5.2 (0.8) $ | 5.2 (0.9) $ | 5.3 (0.7) $ | 5.3 (1.0) $ | 5.3 (0.8) $ | 6.4 (1.5) $ | 6.5 (1.6) $ | 6.5 (1.8) $ | 6.4 (1.5) $ | 6.1 (1.7) $ | <0.001 |
| **Albumin (g/dL)** | 4.5 (0.4) | 4.3 (0.3) | 4.2 (0.4) | 4.2 (0.3) | 4.1 (0.4) | 4.5 (0.4) | 4.4 (0.4) | 4.3 (0.3) | 4.2 (0.3) | 4.1 (0.4) | <0.001 |
| **Creatinine (mg/dL)** | 0.8 (0.2) | 0.7 (0.2) | 0.8 (0.1) | 0.8 (0.2) | 0.8 (0.2) | 0.8 (0.1) | 0.8 (0.1) | 0.8 (0.2) | 0.8 (0.2) | 0.9 (0.2) | <0.001 |
| **eGFR (ml/min/1.73 m2)** | 129 (13.2) | 121 (13.1) | 112 (10.4) | 103 (9.5) | 88 (22.1) | 126 (13.2) | 118 (12.2) | 110 (13.0) | 101 (13.4) | 88 (20.4) | <0.001 |

**Supplementary Table 2 (S2). Descriptive Statistics of Men with and without Dyslipidemia.** Median values and interquartile range (in parentheses) are shown. $ indicates significant difference compared to the same age group according to Dwass-Steel-Critchlow-Fligner pairwise comparisons.* Kruskal-Wallis test.
